# Exploring the Chinese Public’s Perception of Omicron Variants on Social Media: LDA-Based Topic Modeling and Sentiment Analysis

**DOI:** 10.3390/ijerph19148377

**Published:** 2022-07-08

**Authors:** Han Wang, Kun Sun, Yuwei Wang

**Affiliations:** School of Journalism and Communication, Jinan University, Guangzhou 510632, China; jacobwang0606@163.com (H.W.); kunsun_123@163.com (K.S.)

**Keywords:** social media, omicron, COVID-19, topic modeling, sentiment analysis

## Abstract

The COVID-19 pandemic caused by SARS-CoV-2 is still raging. Similar to other RNA viruses, SARS-COV-2 is constantly mutating, which leads to the production of many infectious and lethal strains. For instance, the omicron variant detected in November 2021 became the leading strain of infection in many countries around the world and sparked an intense public debate on social media. The aim of this study is to explore the Chinese public’s perception of the omicron variants on social media. A total of 121,632 points of data relating to omicron on Sina Weibo from 0:00 27 November 2021 to 23:59:59 30 March 2022 (Beijing time) were collected and analyzed with LDA-based topic modeling and DLUT-Emotion ontology-based sentiment analysis. The results indicate that (1) the public discussion of omicron is based on five topics, including omicron’s impact on the economy, the omicron infection situation in other countries/regions, the omicron infection situation in China, omicron and vaccines and pandemic prevention and control for omicron. (2) From the 3 sentiment orientations of 121,632 valid Weibo posts, 49,402 posts were judged as positive emotions, accounting for approximately 40.6%; 47,667 were negative emotions, accounting for nearly 39.2%; and 24,563 were neutral emotions, accounting for about 20.2%. (3) The result of the analysis of the temporal trend of the seven categories of emotion attribution showed that fear kept decreasing, whereas good kept increasing. This study provides more insights into public perceptions of and attitudes toward emerging SARS-CoV-2 variants. The results of this study may provide further recommendations for the Chinese government, public health authorities, and the media to promote knowledge about SARS-CoV-2 variant pandemic-resistant messages.

## 1. Introduction

At present, the COVID-19 pandemic caused by SARS-COV-2 is still raging. It is normal for viruses to change and evolve over time since they spread from person to person. The above changes are termed variants when they are significantly different from the original virus [1]. Similar to other RNA viruses, SARS-COV-2 mutates as it spreads, producing many variants. These variants exhibit high transmissibility, and the ability of the variants to evade immunity threatens global efforts to control the pandemic. Some variants have very high transmissibility, such as the delta variant, which has been shown to be 40% to 60% more transmissible than the alpha variant [2], while others alter the antigenicity of the virus to evade immunity from vaccination or prior infection, such as the beta [3] and gamma [4] variants. On 9 November 2021, the new variant B.1.1.529 was first detected in South Africa [5], designated as a variant of concern by the World Health Organization, and named omicron [6]. Although preliminary evidence suggested that omicron infections are 91% less lethal and 51% less likely to require hospitalization than delta [7], because omicron spreads over a short period of generations and is highly variable in antigenicity [8], it is more infectious than previous strains [9]. This led to its rapid spread to many countries and regions around the world, with global cases increasing exponentially. The omicron subvariant BA.2 accounts for nearly 86% of all sequenced cases and has become the dominant strain globally [10] according to the World Health Organization.

With the rapid global spread of omicron, China is also facing the challenge of dealing with this variant. On 13 December 2021, the first case of the omicron variant infection in mainland China was detected in Tianjin (in northern China). Subsequently, omicron infection cases were found in several cities in China. Since then, social media, including Sina Weibo, have sparked heated discussions about omicron. The widespread use of social media has accelerated the process of exchanging information and expressing opinions on public events and health crises [11,12]. During the COVID-19 pandemic, social media became an important platform for the public to discuss information related to COVID-19. Many investigators have conducted informational epidemiological studies by collecting social media data. Ashish Kumar et al. conducted a sentiment analysis of COVID-19-related tweets on Twitter to gain insights into social media’s perception of COVID-19-related information [13]. Abd-alrazaq et al. conducted an information-monitoring study of COVID-19-related tweets on Twitter to explore the main topics and emotions of discussion related to the disease [14]. Junze Wang et al. conducted topic modeling and emotional orientation analysis of COVID-19-related posts on Sina Weibo [15]. The above findings obtained through topic modeling or sentiment analysis can track diseases and assess public awareness of health problems, thus realizing disease prediction and providing a source of advice for relevant government agencies and health departments to respond to public concerns [16].

With the continuous variation of SARS-COV-2 and the continuous development of the public discussion on social media, it is worth continuing to collect and analyze the big data from social media to track and understand the public’s opinions and emotional response to the virus variants. Based on existing studies, public opinions expressed on Sina Weibo were analyzed using topic modeling and sentiment analysis.

The aim of this study was to answer three questions as follows:

(1) What are the topics of public opinions about omicron based on social media posts?
(2) What emotional orientations and specific emotions do the public have toward omicron?
(3) Have the public emotions toward omicron changed over time?

This study has three main contributions. First, to the best of our knowledge, there is still a lack of information on epidemiological studies on the SARS-COV-2 variants. Based on existing studies, public discussions and emotions were further tracked and analyzed through big data mining and analysis, and knowledge was contributed to information epidemiology. Second, the results of topic modeling and discussion can help us understand Chinese social media users’ concerns about the SARS-CoV-2 variants, which shows certain particularity compared with existing studies. The Chinese public’s concerns about economic impact, vaccine effectiveness, stigma and other issues should be solved by the government; otherwise, public trust may be compromised. Finally, government policies should win public support to better achieve their goals. To gain insights into the public mood and how it changes, it is imperative for decision-makers to make suitable interventions during impending outbreaks. Our study helps to understand the public mood and how it changes.

## 2. Materials and Methods

### 2.1. Data Collection

**Figure 1:**
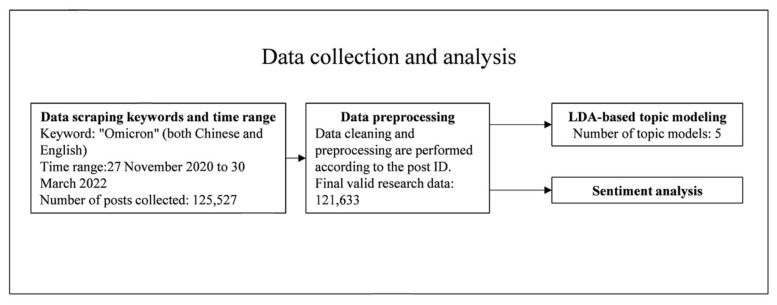
The research design.

In this study, Sina Weibo was selected as the data collection object. Sina Weibo, China’s leading social media user platform, allows users to send and receive posts with short character limits and retrieve text content by searching for specified keywords within a defined date range [17]. Although the B.1.1.529 variant was detected on 9 November 2021, the public debate was sparked only after the World Health Organization named the variant. Therefore, this study started at the time when the omicron variant was first publicly discussed on Weibo (0:00 27 November 2021). On 31 March 2022, we noticed that the discussion of the new variant appeared on Weibo, and the discussion of the omicron variant decreased; omicron variants can be divided into many subvariants (e.g., BA.1, BA.1.1, and BA.2). However, on social media, people usually do not discuss individual subvariants but use the common term “omicron” to describe them. Therefore, Python programming language scripts and Weibo open Application Programming Interface (API) were used to search for the keyword “omicron” (both Chinese and English). The data crawling time range was from 0:00 on 27 November 2020 to 23:59:59 on 30 March 2022 (Beijing time), and a total of 125,527 Weibo posts about omicron were collected. It is worth noting that the start time for the data acquisition for this article was 27 November 2020 and that data from before this time are not within the scope of this study. To avoid repeated data, we cleaned the data according to Weibo ID and finally obtained 121,633 posts as valid data for this study. For each Weibo post, we collected and downloaded the following features: (1) the full text, (2) the post time, (3) the post ID and (4) the users’ description/self-created profile. Figure 1 shows the overall research design framework of this study.

### 2.2. LDA-Based Topic Modeling

One of the aims of this study was to understand what Chinese social media users have been discussing about the omicron variant, so latent Dirichlet allocation (LDA) topic modeling was used to gain an overall view of topic distribution [18]. In addition, the optimal number of topics was obtained based on the perplexity indicator. LDA topic modeling is an unsupervised learning algorithm that has been widely used in social media topic recognition [19]. It can give the topic of each document in the dataset in the form of probability distribution, and it does not need manual marking in the training process. In this study, the data were preprocessed from the acquired dataset. First, to ensure the reliability and good identifiability of the data, we used the Python (The packages included in Anaconda 5.3.0 for 64-bit Windows with Python 3.6, https://docs.anaconda.com/anaconda/packages/old-pkg-lists/5.3.0/py3.6_win-64/, accessed on 20 November 2021) jieba word segmentation software package (Jieba 0.42.1 version, https://github.com/fxsjy/jieba, accessed on 20 November 2021, Beijing, China, Creator: Sun Junyi) to segment the obtained dataset and established a glossary of words no longer in use. The glossary comprises common modal words, auxiliary words, meaningless network terms, etc. In this study, most of the noisy words were removed using the word list, which played an optimization role in the subsequent topic modeling. LDA topic extraction was carried out using Gensim, a machine learning package based on Python language, and the perplexity index and document similarity were calculated by Python programming. The basic idea of perplexity is to assign a language model with a high probability to the test set [20]. As revealed by the smaller value of perplexity, the model is capable of effectively predicting the new text; perplexity generally decreases with the increase in the number of potential topics. In the process of determining the number of topics, continuous debugging was conducted for 15 topics. After the number of topics was constantly adjusted, the perplexity score reached its optimal value when the number of topics was five; therefore, the number of topic models in this study was set as five. Meanwhile, when the LDA model gives the result of the topic model, it will provide the topic word under the related topic and the weight of the topic word under the subordinate topic (in the Appendix A, the top 20 subject terms output by the LDA model when the number of subjects is 5 are shown). Under each topic, 10 high-probability topic words with higher weights were selected and presented as the results for related topics. Meanwhile, topic names were based on the common content presented by high-probability topic words. To give a meaningful explanation of the subject, we used partial text from microblog posts as elementary context units [21] (i.e., significant parts of the text as defined by word co-occurrences).

### 2.3. Sentiment Analysis

Since Weibo is a social platform mainly composed of short texts, we conducted our text sentiment analysis under different granularity according to the emotional orientations of words provided by the sentiment dictionary. In the selection of tools, we chose the DLUT Emotion Vocabulary Ontology database (DLUT) as the tool for the emotion orientation and sentiment analysis of this dataset. DLUT is a Chinese ontology resource organized and marked by the Information Retrieval Laboratory of Dalian University of Technology. It refers to Modern Chinese Dictionary, A Dictionary of Chinese Praise and Blame Words, Chinese Adjective Dictionary and other authoritative Chinese dictionaries and contains about 30,000 emotion words. The tool includes Chinese words or short parts of speech types, emotional categories, emotional intensity and polarity, and it has high coverage and accuracy for Chinese words. It has been widely used in sentiment analysis of Chinese texts [22]. The emotion classification system of DLUT Chinese emotion word ontology is constructed on the basis of Ekman’s influential six categories of emotion classification [23]; specifically, the lexical ontology adds the emotion category “good” to make a more detailed division of commendatory emotion. DLUT divides the emotional intensity of words into five levels (1, 3, 5, 7 and 9). The higher the level, the stronger the emotional intensity. DLUT provides seven basic emotions, including good, happy, surprise, disgust, fear, anger and sadness, as well as a specific emotion score for each post. According to the affective score, emotions are categorized into three polarities: negative, positive and neutral. Posts with an emotion score less than 0 were judged as negative, those with an emotion score greater than 0 were judged as positive, and those with an emotion score equal to 0 were judged as neutral. Table 1 describes how DLUT defines emotional polarity and the seven emotional categories. As part of the data processing, we matched the dataset of this study with the ontology library of DLUT emotion words based on Python language. On this basis, the specific matching score of seven basic emotions and the overall emotional score of each post were obtained, and the emotional polarity of the post was determined.

## 3. Results

### 3.1. Omicron–Related Topics

Table 2 highlights the most frequent terms in the case of the five topics.

#### 3.1.1. Topic 1: Omicron’s Impact on the Economy

Topic 1 includes words related to the economic impact of the emergence of the omicron variants, especially “index”, “inflation” and other specific words related to the economy. Users reported concerns and fears about the negative economic impact of omicron’s pandemic. For example, in the early stages of omicron’s detection, users engaged in discussions about the economic situation. Users believed omicron was spreading faster than the previous SARS-COV-2 variant and would hit global growth harder and stoke inflation further. “Gold”, “crude” and “dollar” are all words related to financial markets, and omicron’s impact on the economy can be judged by comparing currencies, commodities and other indicators.

#### 3.1.2. Topic 2: Omicron Infection Situation in Other Countries/Regions

Topic 2 reflects public concern in China about omicron variant infection in other regions/countries of the world. “The United States”, “Hong Kong”, “United Kingdom”, and “Japan” are all words that represent countries/regions. In terms of the weight of words, the Chinese public has had the most discussion about omicron infection in the above countries/regions. Omicron was detected in China later than in the countries mentioned above. The spread of the omicron variant in other countries and regions made Chinese users perceive the threat of the high spread of the variant. For example, users looked at daily diagnoses in other countries and omicron’s basic reproduction number R0. Then, omicron variants were detected in China. Chinese users compare omicron’s spread in their country with that of other countries.

#### 3.1.3. Topic 3: Omicron Infection Situation in China

Topic 3 contains words related to omicron variant infection in China. For example, “cases of disease”, “diagnosis”, “outbreak” and “asymptomatic” are used to describe the spread of the omicron variant in China. Since the variant was detected in China, cases of omicron infection have been reported in several Chinese cities. The Chinese government releases daily updates on the number of COVID-19 infections through the media, and this information also appears on social media. For example, users express their concerns about omicron through data such as the number of confirmed cases and asymptomatic cases.

#### 3.1.4. Topic 4: Omicron & Vaccines

The keyword of topic 4 is “vaccines”, including such words as “vaccination”, “transmission” and “research”. Developing a vaccine that builds up the body’s immune system is one of the key methods of fighting the pandemic [24], but the immune evasion characteristic exhibited by the omicron variant requires constant updating to improve its effectiveness. There has been a heated public debate about vaccines in China. On the one hand, Chinese users are concerned about the effectiveness of existing COVID-19 vaccines against the newly emerging omicron variant, especially whether the existing COVID-19 vaccines can prevent severe symptoms after infection with omicron. On the other hand, Chinese users are paying close attention to the latest developments in omicron-specific vaccines, and they are looking forward to the early launch of the latest vaccine.

#### 3.1.5. Topic 5: Pandemic Prevention and Control for Omicron

The keywords of topic 5 are related to pandemic prevention and control. For example, “nucleic acid test”, “facemask” and “controls” are used to describe how the Chinese government has faced the challenge of the omicron variant. As the omicron variant has been found around the globe and imported cases have been reported in Chinese cities, the Chinese government has stepped up testing through a series of measures. Notably, facemask and SARS-COV-2 nucleic acid testing have become an important part of daily life for the Chinese public. For example, users mention how often they take the nucleic acid test each week and how they feel about being tested.

### 3.2. Results of Sentiment Analysis

Based on the DLUT-Emotion lexicon ontology, we examined the three emotional directions of 121,632 valid Weibo posts. Among them, 49,402 posts were judged to be positive, accounting for 40.6%; 47,667 posts were considered negative, accounting for 39.2%; and 28,643 posts were considered neutral, accounting for about 20.2%. Sentiment attribute analysis shows that good is the most common emotion in the corpus (35.42%), followed by fear (29.9%), happy (24.8%) and disgust (9.2%). Less common emotions observed were sadness (1.6%), surprise (0.2%) and anger (0%).

To observe the temporal variations in public sentiment, we divided the selected microblogs into four periods (each period was 31 days): 27 November to 27 December 2021, 28 December 2021 to 27 January 2022, 28 January to 27 February 2022 and 28 February to 30 March 2022. We found that the public’s emotional orientation changed over time (Table 3): From the first stage to the third stage, the proportion of microblog words with positive emotions showed a significant upward trend, while the proportion of microblog words with negative emotions showed a downward trend. From stage one to stage four, fear continued to decline while good continued to rise.

## 4. Discussion

The main purpose of this study was to explore topics and emotions related to omicron based on the discussion content generated by online users of Sina Weibo. A total of 121,632 pieces of omicron data were collected on Sina Weibo from 0:00 on 27 November 2021 to 23:59:59 on 30 March 2022. Topic model analysis was conducted based on LDA, and sentiment analysis was conducted based on Dlut-Emotion ontology on the above data. Three valuable findings emerged from this study: (1) Public discussion of omicron can be divided into five topics: omicron’s impact on the economy, the omicron infection situation in other countries/regions, omicron infection situation in China, omicron and vaccines, and pandemic prevention and control for omicron. (2) The results of sentiment analysis showed that both positive and negative emotions accounted for large proportions. Good was the most common emotion in the corpus, followed by fear, happy and disgust. Less common emotions observed were sadness, surprise and anger. (3) The time trend analysis of emotional attribution showed that fear was decreasing and good was increasing.

### 4.1. Results of Topic Modeling

Modeling the topics of the Weibo posts helps us understand the focus of the public discussion about omicron. The results showed several key points. First, topic 3 (omicron infection situation in China) and topic 5 (pandemic prevention and control for omicron) are consistent with previous research on COVID-19 [25]. Previous studies have also shown that prevention and control procedures, including isolation, as well as confirmed cases and medical reports, have been major topics of discussion in past outbreaks [26]. It is therefore revealed that public concern about these issues has changed little from the early days of the COVID-19 outbreak to the omicron pandemic. The above issues are not only at the heart of public concern and discussion; they highlight that governments and public health departments need to respond to public concerns. Second, we found several topics that were new since previous studies: topic 1 (omicron’s impact on the economy), topic 2 (omicron infection situation in other countries/regions) and topic 4 (omicron and vaccines). Previous research has reported on the political topics of COVID-19 in Twitter discussions [27], and we found that the public is more concerned about the economic impact of a new variant of SARS-COV-2 on Chinese social media. On the one hand, the COVID-19 pandemic did have a serious negative impact on the economy, which is reflected in the impacts of COVID-19 on the supply and demand in the global economy, which has resulted in a global economic recession [28]. As a result, it is understandable that after omicron’s appearance, the Chinese public expressed concern via social media that the economic situation could get worse. On the other hand, we believe this may be correlated with the Chinese government’s censorship of the media, with research suggesting that continued censorship of political content means that most online services in China are focused on entertainment [29]. As a result, public discussion of the new variant of SARS-COV-2 has focused on economic topics, not politics. Topic 2 suggests that the Chinese public is highly concerned about omicron infection in other countries and regions, which indicates significant information gaps between countries/regions in response to the omicron pandemic. Taking advantage of the time lag to disseminate scientific information about new strains of the virus could help reduce public panic. Topic 4 (omicron and vaccines) is also a new topic of this study. The findings on this topic are significant as they show that the idea that COVID-19 can be resisted through vaccination has been accepted by a large part of the Chinese public. The data on vaccination in China also prove this. As of 31 March 2022, 90.63% of China’s total population had been covered by vaccines, and 88.11% had been fully vaccinated [30]. Of course, we also need to be mindful of discussions about the effectiveness of existing vaccines against new virus variants. Extensive analysis of scientific findings related to COVID-19 vaccine research remains essential to helping individuals accurately understand vaccine efficacy [31].

### 4.2. Overall Positive Public Sentiment to Omicron Variants

The results of the emotional orientation analysis indicate that Weibo users show obvious emotions to omicron. Only 20% of posts were judged to be neutral, while the percentage of posts judged to be positive was very close to that of posts judged to be negative, about 40%. The public attitudes reflected by positive and negative emotional orientations were further discussed based on specific texts. Among the data judged to be positive, many posts expressed support for “fighting the pandemic”. Since the omicron variant was detected in China, cases of omicron infection have been reported in Tianjin, Guangzhou, Shenzhen, Shanghai and other Chinese cities. Many Weibo users have posted their support for cities with omicron cases. For example, Tianjin was the first city in China to detect omicron cases, and many users have used Weibo to pray and wish for the residents of Tianjin. In addition, there were many posts expressing the Chinese public’s confidence that the COVID-19 pandemic will eventually be defeated. Even in the face of the challenge of the new variant of SARS-COV-2, users expressed their pride in China’s anti-pandemic policies by forwarding media posts. For instance: “China’s dynamic zero-COVID policy can cope with omicron’s challenge”. In the data judged as negative emotions, the negative emotions of the public mainly target two aspects. On the one hand, negative sentiment is directed at other countries and regions. Some users are concerned about omicron infections abroad. For example, “Nearly three-quarters of new cases in the US are omicron” and “The number of new infections in Japan exceeds 70,000”. A significant number of users attributed the increase in omicron infections abroad to the lack of strict quarantine measures as in China. For example, one user posted that “The number of confirmed cases in Europe and the US is reaching new heights in the face of omicron”. In addition, Chinese news reports suggested that some patients in China had contracted omicron from exposure to goods from abroad, which also created negative feelings among users. The omicron outbreak is believed to have originated abroad. Stigma theory argues that stigmas can serve as the cognitive basis of social grouping; psychological groups are the underlying nature of identifiable social groups that individuals engage with through a mutual acceptance and adherence to defined ingroup–outgroup categories [32]. Previous research has shown that the COVID-19 (or SARS-CoV-2) pandemic has created an environment rife with stigmatization [33]. The rise of stigmatization and prejudice toward groups (particularly Asian) occurred as attempts increased to place blame for this phenomenon on those associated as carriers of the virus [34]. The results of this study indicate that stigmatization also exists on social media in China and that stigmatization reinforces cognitive differences between in-group and out-group members. We believe that negative sentiment posts directed at other countries and regions, like the previous “Chinese virus” incidents, may increase the level of stigma associated with COVID-19 and undermine efforts to address the COVID-19 pandemic [35]. On the other hand, many users expressed concern and anxiety about the dangers of the omicron variant itself. Many posts expressed concern that existing vaccines might not work against omicron. One post, for example, said: “There are no vaccines in the world against omicron. If you don’t get vaccinated, your immune system may not be damaged and you may not have ADE reactions”. Previous studies have determined that anti-vaccine messages often revolve around potential side effects, adverse reactions to vaccines, misinformation and conspiracy theories [36]. Our results also confirm that anti-vaccine messages remain in the face of the SARS-COV-2 variant.

### 4.3. Changes in Specific Emotional Connotations

The cognitive physiological theory of emotion holds that the production of emotion is affected by three factors: environmental events, physiological conditions and cognitive processes. The cognitive process is the key factor in determining the nature of emotion [37]. The cognitive physiological theory of emotion provides a theoretical explanation path to understanding the process of emotional change. Specific to this study, from the perspective of changes in specific emotions, changes in positive emotions reflect an increase in people’s confidence in the face of omicron variants. Changes in negative emotions reflect changes in people’s perceptions of omicron, which arise from the deepening public understanding of omicron variants. In the sentiment analysis, words judged to be good appeared the most frequently and increased over time, thus revealing that the increasing number of omicron infections has not affected the Chinese public’s confidence in defeating omicron. The posts related to good praise and affirm China’s anti-pandemic policies. The Chinese public considers that the COVID-19 pandemic can be stopped as long as the local governments implement strict zero-COVID policy, which is highly consistent with China’s current national conditions. In addition, feelings of fear decreased over time. We believe the above result may be related to the increasing awareness of the emerging omicron variant among the Chinese public. The knowledge gap hypothesis holds that as time goes by, people are exposed to increasing amounts of media information and acquire increasing knowledge in specific fields [38]. In the early days, when news of the detection of a new SARS-CoV-2 variant was reported in the media, people were terrified of the new variant because they did not know its characteristics. However, as research into the new SARS-CoV-2 variants deepened and scientific reports about omicron increased on social media, the word “fear” appeared less frequently, which is consistent with common sense. This is yet another example of the potential psychological benefits that governments and health officials can bring to the public by providing accurate information about the pandemic and reducing the impacts of misinformation on public sentiment [39].

### 4.4. Limitations and Future Research

However, despite these valuable findings, there are still some limitations of this study. First, omicron was the only keyword for mining the data, and the results obtained may be incomplete. Future research can expand keywords to enhance the representativeness of data. Second, this study only obtained data from Weibo, which is only one of the sources of information about omicron. Future research can expand data sources to include other social media and even other media channels. Third, young people account for a very large proportion of Weibo users. Therefore, the results and significance of this study are more applicable to young groups. Future surveys on the perceptions of other age groups can be carried out through questionnaires, in-depth interviews and other methods. Finally, since this article is a Brief Report, the article still lacks theory to some extent. Future research can further strengthen the theoretical interpretations and enhance the theoretical significance of the article.

## 5. Conclusions

Using a large sample of microblog data for informational epidemiology, this study explored public discussion and sentiment related to omicron. Our results provide insights into the public discussion and concerns of Chinese Weibo users about the novel SARS-CoV-2 variant omicron between 27 November 2021 and 30 March 2022. We have identified not only topics for discussion that have been confirmed by existing research, i.e., vaccination measures and the infection situation, but also new topics for discussion: omicron’s impact on the economy, the omicron infection situation in other countries/regions and omicron and vaccines. The results of the sentiment analysis helped us gain insights into the new SARS-CoV-2 variant’s public concern about vaccine effectiveness and the stigmatization associated with COVID-19. The results presented here also confirmed the need for timely and accurate information on social media to improve the mental health of the public. Real-time monitoring and assessment of social media users’ discussions and concerns can be beneficial to understanding the public’s real concerns, informing public health emergency response and providing effective advice to improve public mental health and prepare for similar incidents in the future.

## Figures and Tables

**Table 1:** DLUT Sentiment Polarity and Attribute Word Examples.

Class	Item	Sample Words
**Sentiment attribution**	good	excellent, trust, respectful, strictly, outstanding
	happy	happy, joy, expectation, excited, fun
	surprise	surprising, shocked, strange, suddenly, happen
	disgust	shameful, hate, vanity, hypocrisy, dirty
	fear	tense, flustered, afraid, blankly, helpless
	anger	angry, furious, peeved, fury, roar
	sadness	painful, depressed, failure, suffering, guilt
**Sentiment polarity**	Negative	frantic (1), profit-making (3), fraud (5), buy off (7), desperate (9)
	Positive	opportunity (1), innovation (3), outstanding (5), reliable (7), elite (9)
	Neutral	relax (1), clear (3), suggestion (5), safe (7), desperately (9)

**Table 2:** Topics extracted from Weibo related to omicron.

Topic Number	Description of Extracted Topics and Words
Topic 1:	**Omicron’s impact on the economy**
	Market, economy, impact, index, dollar, rise, crude, inflation, oil price, gold, the Federal Reserve System,
Topic 2:	**Omicron infection situation in other countries/regions**
	United States, pandemic situation, infection, diagnose, Hong Kong, United Kingdom, Japan, South Korea, France, Europe
Topic 3:	**Omicron infection situation in China**
	Cases of disease, diagnosis, infected people, outbreak, asymptomatic, mainland, cumulative, variant, newly increased, detection
Topic 4:	**Omicron & Vaccines**
	Omicron, variants, COVID-19, vaccine, transmission, vaccination, research, experts, serious illness, response, mutations,
Topic 5:	**Pandemic prevention and control for Omicron**
	Epidemic, prevention and control, nucleic acid test (NAT), facemask, work, measures, control, protective, community, quarantine

**Table 3:** Results of sentiment orientation and attribute analysis.

Stage	Sentiment Orientation	Volume	Proportion	Sentiment Attribute	Volume	Proportion
Stage1 (27 November–27 December)	positive	13,904	34.06%	good	12,546	30.76%
				happy	9454	23.18%
	neutral	8554	20.98%	surprise	59	0.14%
				disgust	4018	9.85%
	negative	18,335	44.96%	fear	13,944	34.18%
				anger	0	0%
				sadness	772	1.89%
	Total	40,793	100.00%	Total	40,793	100.00%
Stage2 (28 December–27 January)	positive	14,682	39.58%	good	12,441	33.54%
				happy	10,068	27.14%
	neutral	8239	38.21%	surprise	64	0.17%
				disgust	3860	10.40%
	negative	14,177	22.21%	fear	10,225	27.56%
				anger	0	0%
				sadness	440	1.19%
	Total	37,098	100.00%	Total	37,098	100.00%
Stage3 (28 January–27 February)	positive	11,094	50.44%	good	9063	41.20%
				happy	5303	24.11%
	neutral	3411	15.50%	surprise	54	0.25%
				disgust	1476	6.71%
	negative	7491	34.06%	fear	5879	26.73%
				anger	0	0%
				sadness	221	1.00%
	Total	21,996	100.00%	Total	21,996	100.00%
Stage4 (28 February–30 March)	positive	9722	44.71%	good	9031	41.53%
				happy	5367	24.68%
	neutral	4359	20.04%	surprise	54	0.25%
				disgust	1872	8.61%
	negative	7664	35.25%	fear	5152	23.70%
				anger	0	0%
				sadness	269	1.23%
	Total	21,745	100.00%	Total	21,745	100.00%

## Data Availability

The data presented in this study are available on request from the corresponding author, Y.W., at twyw1218@jnu.edu.cn. The data are not publicly available in accordance with funding requirements and participant privacy.

## Appendix A

**Table A1:** The weight of the topic word.

Topic 1: Omicron’s Impact on the Economy	Weight	Topic 2: Omicron Infetion Situation in Other Countries/Regions	Weight	Topic 3: Omicron Infection Situation in China	Weight	Topic 4: Omicron & Vaccines	Weight	Topic 5: Pandemic Prevention and Control for Omicron	Weight
Market	0.013	The United States	0.015	Cases of disease	0.055	Omicron	0.046	Epidemic	0.04
Economy	0.01	Pandemic situation	0.015	Diagnosis	0.032	Variants	0.028	The prevention and control	0.029
Impact	0.007	Infection	0.014	Infected People	0.032	COVID-19	0.023	Nucleic acid testing (NAT)	0.015
Indexes	0.007	Diagnose	0.014	Outbreak	0.028	Vaccine	0.022	Detection	0.013
Expectations	0.006	Hong Kong	0.013	Omicron	0.024	Transmission	0.02	Health	0.01
Dollar	0.006	COVID-19	0.013	Asymptomatic	0.021	Infection	0.017	Facemask	0.01
Epidemic	0.006	Virus	0.01	Mainland	0.018	Strain	0.016	Omicron	0.009
Demand	0.006	Newly increased	0.009	Cumulative	0.018	Vaccination	0.009	Work	0.009
Global	0.005	United Kingdom	0.008	Variants	0.017	Research	0.009	Epidemic prevention workers	0.009
Rising	0.005	Variants	0.007	Newly increased	0.016	Epidemic	0.008	Measure	0.007
Price	0.005	Omicron	0.006	Positive	0.015	Expert	0.006	Control	0.006
Growth	0.005	Vaccinate	0.006	Detection	0.015	China	0.006	Protective	0.005
Crude	0.004	Death	0.006	Report	0.014	Global	0.006	Community	0.005
Expected	0.004	Japan	0.005	COVID-19	0.013	Serious illness	0.005	Gather	0.005
Inflation	0.004	South Korea	0.005	Pneumonia	0.011	Severe case	0.005	Risk	0.005
Oil price	0.004	Strain	0.005	Quarantinen	0.011	Response	0.004	Vaccinate	0.005
Gold	0.004	Immigration	0.005	Nucleic acid testing (NAT)	0.01	Mutations	0.004	Area	0.004
Decline	0.003	France	0.003	Virus	0.009	Data	0.003	Place	0.004
The Federal Reserve System	0.003	Europe	0.002	Infection	0.008	Infectiousness	0.003	Cooperate	0.004
Policy	0.003	South Africa	0.002	Chinese Center for Disease Control and Prevention	0.006	Immune	0.003	Quarantinen	0.004
